# Scaling up public health interventions: case study of the polio immunization program in Indonesia

**DOI:** 10.1186/s12889-021-10647-6

**Published:** 2021-03-29

**Authors:** Utsamani Cintyamena, Luthfi Azizatunnisa’, Riris Andono Ahmad, Yodi Mahendradhata

**Affiliations:** 1 Center for Tropical Medicine, Faculty Medicine, Public Health, and Nursing, Universitas Gadjah Mada, Yogyakarta, Indonesia; 2 Department of Health Behavior, Environment, and Social Medicine, Faculty of Medicine, Public Health and Nursing, Universitas Gadjah Mada, Yogyakarta, Indonesia; 3 Department of Biostatistics, Epidemiology, and Population Health, Faculty of Medicine, Public Health and Nursing, Universitas Gadjah Mada, Yogyakarta, Indonesia; 4 Department of Health Policy and Management, Faculty of Medicine, Public Health and Nursing, Universitas Gadjah Mada, Yogyakarta, Indonesia

**Keywords:** Scaling up, Polio, Immunization, Coverage, Public health intervention, Indonesia

## Abstract

**Background:**

The scaling up of public health interventions has received greater attention in recent years; however, there remains paucity of systematic investigations of the scaling up processes. We aim to investigate the overall process, actors and contexts of polio immunization scaling up in Indonesia from 1988 until 2018.

**Methods:**

A mixed method study with sequential explanatory design was conducted. We carried out a quantitative survey of 323 actors involved in the polio program at national and sub-national levels, followed by Key Informant Interviews (KII)s. Document review was also carried out to construct a timeline of the polio eradication program with milestones. We carried out descriptive statistical analysis of quantitative data and thematic analysis of qualitative data.

**Results:**

The scaling up of polio immunization in Indonesia started as a vertical expansion approach led by the Ministry of Health within a centralized health system. The coverage of immunization increased dramatically from 5% in the earlier 80s to 67.5% in 1987; incremental increases followed until achieving Universal Child Immunization (UCI) in 1990 and subsequently 95% coverage in 1995. Engagement of stakeholders and funding made the scaling up of polio immunization a priority. There was also substantial multisector involvement, including institutions and communities. Local area monitoring (LAM) and integrated health posts (*Posyandu*) were key to the polio immunization implementation strategy. Challenges for scaling up during this centralized period included cold chain infrastructure and limited experience in carrying out mass campaigns. Scaling up during the decentralized era was slower due to expansion in the number of provinces and districts. Moreover, there were challenges such as the negative perception of immunization side-effects, staff turnover, and the unsmooth transition of centralization towards decentralization.

**Conclusion:**

Vertical scaling up of polio immunization program intervention was successful during the centralized era, with involvement of the president as a role model and the engine of multi sector actors. *Posyandu* (integrated health posts) played an important role, yet its revitalization after the reform-decentralization era has not been optimum.

## Background

Research interventions are often carried out on a small scale or within a particular context due to time and resource limitations. The results of small study projects are thus often difficult to apply in a complex health system; however, expansion efforts could potentially be executed effectively on a large scale [1]. The process of transferring this evidence into practical large-scale implementation is usually known as “scaling up”. Through implementation research, scaling up can address the existing gap of knowledge and practices [2].

Even though the term scaling up is not new in health systems research, there is still no widely agreed definition. The definition of scaling up varies from a stage of implementing the result of laboratory trials to a human or community; to widening the result of a pilot study to a broader setting and context [3]. In this paper, the definition is taken from WHO/ExpandNet, i.e., *“deliberate efforts to increase the impact of successfully tested health innovations so as to benefit more people and to foster policy and program development on a lasting basis”* [4].

Barker, Reid and Schall (2016) proposed four steps that should be fulfilled in scaling up, i.e., introducing and testing the intervention that will be taken to full scale as the first step, followed with the development of the scalable unit as the second step. The next steps are to test the response in a setting that will represent the full scale context, before then going to full scale; this often unfolds rapidly to enable a more significant number of sites or divisions to adopt and/or replicate the intervention [5].

The frameworks for scaling up are also as diverse as the definitions of it [5–8]. Many frameworks dedicated to scaling up have been developed to help transform health research interventions into pathways towards achieving millennium development goals. As an example, the Institute for Healthcare Improvement defined several important elements in the framework, such as: leadership responsibility, packaging the new ideas, communication, social system strengthening, measurement and feedback, and knowledge measurement. The proposed structure by Yamey (2011) emphasized key factors that include: choosing a simple intervention that is widely agreed to be valuable, strong leadership and governance, active engagement of a range of implementers and the target community, tailoring the scale-up approach to the local situation, and incorporating research into implementation. Additionally, the WHO/ExpandNet framework formulates five elements listed as: environment, innovation, resource team, user organization, and scaling up strategy [4, 9].

In order to contribute to the emerging body of knowledge on scaling up, we investigated the scaling up of a polio eradication program in Indonesia. Polio immunization started in the country with very low coverage, then increased gradually, and in 2014 Indonesia received the polio-free certificate from South East Asia Region Office (SEARO). Similarly to other settings in low-and middle-income countries (LMIC) s, Indonesia lacks the resources, systems, and infrastructure required to prevent infectious diseases and potential outbreaks [10]. We will thus analyze how the polio program was scaled up, actors and the context, the scaling up strategy, and the overall process of polio immunization scaling up.

## Methods

### Study setting

This study was conducted in Indonesia, at national and subnational level. In addition to the national context, this study focused on several provinces including Daerah Istimewa Yogyakarta, West Java, Banten, East Java, Aceh, and East Nusa Tenggara Province; selection was based on recommendation by the MoH in consideration of their experiences in polio eradication program management.

### Design

As part of the Synthesis and Translation of Research and Innovations from Polio Eradication (STRIPE) project (https://stripe.jhu.edu/), we conducted a convergent mixed-method study complemented by document review [11]. We systematically collected documents and reports on polio related information. The quantitative survey was carried out on actors of the polio eradication program, while a qualitative study was carried out on key informants. Both quantitative and qualitative approach were used with concurrent data collection and final integration. All data were collected simultaneously between June 2018 and March 2019.

Document review and qualitative analysis were conducted to describe context and the polio program timeline. The program’s challenges, strategies, and lessons learned were derived from quantitative and qualitative data. We adapted the WHO/ExpandNet framework to explain how immunization coverage in Indonesia was scaled up.

### Data collection and analysis

Grey literature was collected from relevant sources, such as the Ministry of Health (MoH), World Health Organization (WHO) Indonesia, the national library, university libraries, newspapers and online search engines. The data or materials must be in Bahasa Indonesia or English and related to the polio eradication program between 1988 until 2018.

In the survey, informants should have had at least 1-year of experience in the polio eradication program. A total of 323 participants were geographically selected considering regional lessons learned during the implementation of the program. The selection of focus area sampled is recommended of the MoH. Participants were recruited with a snowball sampling technique. The trained enumerator reached out and made an appointment to complete a face-to-face survey. The self-administered online survey was also available for participants outside of the selected area that have been discussed.

Key informants were main actors on polio eradication program, both in national and subnational level. They were identified during an inception meeting with the current stakeholder in the MoH. All identified KII participants were recruited through email or telephone. Participants who agreed to participate scheduled a time for direct interview or audio-recorded telephone interview. Interviews were collected by two research assistants that have been trained for Good Health Research Practice [12]. Within this study, we assure confidentiality of all participants.

The survey questionnaire and semi-structured qualitative interview guide were developed by the STRIPE Project Team at the Johns Hopkins Bloomberg School of Public Health based on the Consolidated Framework for Implementation Research (CFIR) framework [11].

Quantitative data were analyzed descriptively by STATA V.12 software and the qualitative data were interpreted by thematic analysis. Through systematic classification of transcripts, the codes and themes were identified. Verbatim participant quotes are presented to highlight the findings. Each interview was approximately 60–90 min and recorded with a digital recorder. Data were transcribed by an experienced transcriber and checked by the interviewer. Transcripts were then imported into Open Code V4.03 software for coding and analysis. Debriefings within the research team were conducted regularly from the beginning of data collection until the data were saturated.

## Result

### Context of the polio immunization program

Indonesia is the largest archipelago in the world and highly influenced by climate. With more than 240 million people that live in uneven distribution, Indonesia consists of numerous ethnic, cultural and linguistic groups. Indonesia has recently emerged as a middle-income economy with a long history as a low-income country. The political and social landscapes have also been evolving through transition from authoritarianism towards democracy and decentralized reforms.

The Indonesian health system has a combination of public and private providers and consists of public and private financing. The public system was administered within a centralized government system until 1999, following a government-wide decentralization. Since then, the decentralized government system has been responsible for the state-owned system, with the coordination of central, provincial, and district governments. The MoH at the national level is in charge of management for tertiary and specialist hospitals, providing strategic direction, setting standards, enforcing regulation, and ensuring the availability of financial and human resources. Provincial governments are responsible for the management of provincial-level hospitals, technical oversight and monitoring of district health services, and coordination of cross-district health issues within the province. District or municipal governments are responsible for the management of district/city hospitals and local Primary Health Cares (PHC) s and their networking [13].

Consistent with the 1988 declaration of the World Health Assembly goal, polio eradication in Indonesia is carried out under the Expanded Program on Immunization (EPI). The Directorate General of Disease Control and Prevention (DGDC), Health Quarantine (DSHQ), and the MoH through the Sub Directorate of Immunization and Surveillance, have taken full liability of the program, prioritizing polio eradication and the provision of the vast majority of funding for all activities. As a component of the program, Acute Flaccid Paralysis (AFP) surveillance is under the Sub Directorate of Surveillance, while the EPI program is under the Sub Directorate of Immunization. Thus, rather than creating an organization for a polio eradication program in Indonesia, the program has been incorporated by the government within existing institutions [14].

In Indonesia, the Oral Poliovirus Vaccine (OPV) was included during routine immunizations in 1981 [14]. The effort towards eradication began in 1991 and has been fully implemented since 1995. Full implementation is stipulated by the following: the polio eradication program is not only related to polio cases or morbidity related to polio, but also relies on active case finding by Active Flaccid Paralysis (AFP) surveillance; and, the provision of routine and supplementary polio immunization. Although Global Polio Eradication Initiative (GPEI) was not a program priority until 1995, the Indonesian MoH continued to make various efforts to increase the coverage of polio immunization. In the era of centralization, MoH, through DGDC and DSHQ, was the primary stakeholder along with partners from WHO, UNICEF, Rotary, and the CDC along with the other ministries [14]. The *Puskesmas* (primary health care centers) and their networks managed and delivered the basic immunization program, however the program could also be accessed through private providers.

Following global efforts; the strategic planning of polio end game strategy, Indonesia also started to pay attention to poliovirus containment and its certification. Efforts were made to maintain polio-free status by ensuring prevention of potential poliovirus leaks return to the environment after the transition of tOPV to bOPV in 2016. Environmental surveillance is considered an early warning system for wild or vaccine-derived poliovirus in areas with poor AFP surveillance or a high risk of poliovirus circulation. In Indonesia, environmental surveillance began in 2004 in Yogyakarta and was applied national-wide in 2016. The AFP and environmental surveillance were supported by Indonesia’s National Polio Lab Network (NPLN). There are three laboratories as part of NPLN, where it continued to receive yearly accreditation through SEAR and WHO HQ Polio Laboratory Network. Bio Farma, a state-owned enterprise and one of NPLN, is appointed as a national polio reference laboratory and a polio-essential facility (PEF) holding OPV/Sabin-only or WPV.

In this paper, we are focusing on the OPV coverage due to length of the program and assumption of how much lesson learned to draw. We use OPV immunization scoping as representative of polio immunization program in Indonesia.

### Scaling up process in Indonesia

**Fig. 1:**
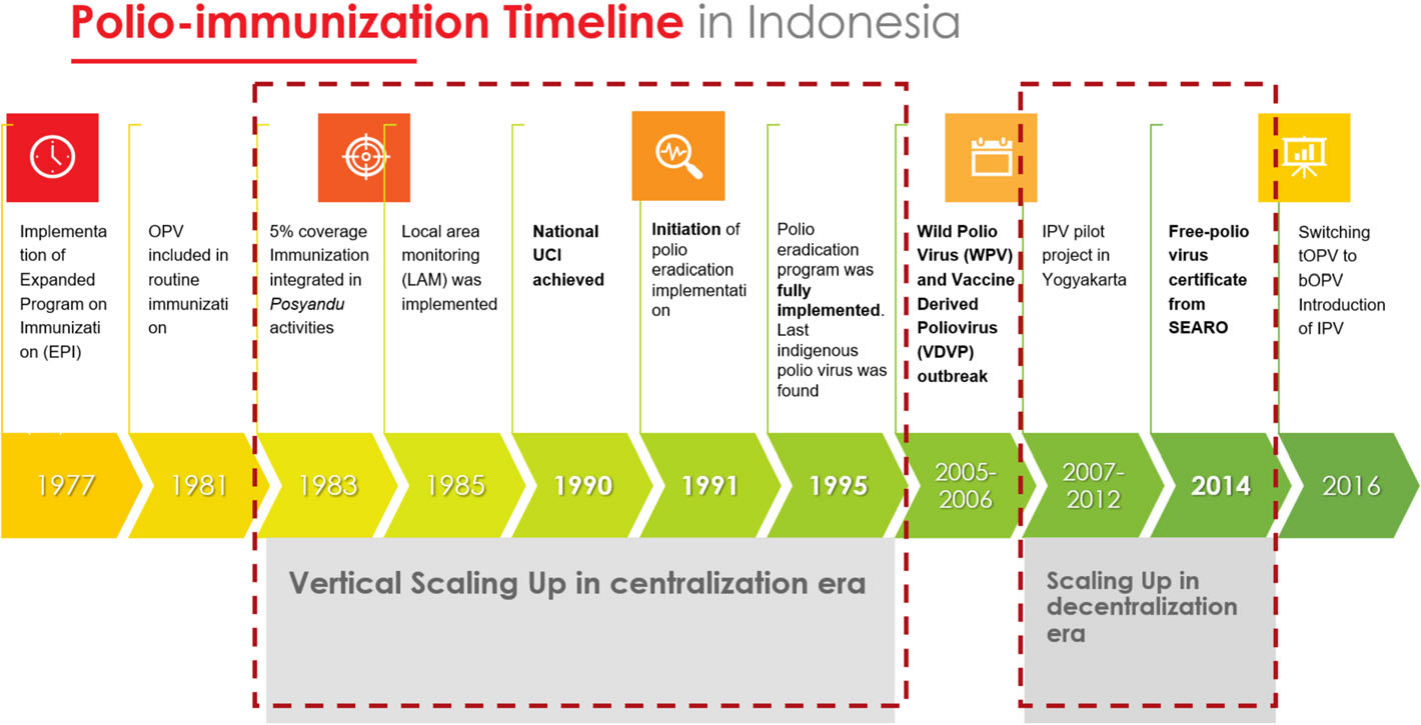
Increasing polio immunization coverage; polio eradication initiative timeframe

Scaling up first appeared in the period of the centralization era, from 1983 to 2000 (Fig. 1). The coverage of immunization increased from 5.8 to 67.5% within 1983–1987, and in 1990 national immunization coverage was achieved. In 1991, polio eradication was initially implemented in Indonesia, and immunization coverage continued to increase. During the period from 1990 to 1995, health allocation increased more than 10 times, and polio immunization was scaled up. In line with this effort, there was a decrease of polio cases from almost 800 cases in 1984 to 24 in 1994 [15]. The indigenous poliovirus was eradicated in Indonesia 4 years after this program was fully implemented in the country. Apart from the outbreak due to imported case in 2005–2006, no polio case had been found in Indonesia until 2018.

**Fig. 2:**
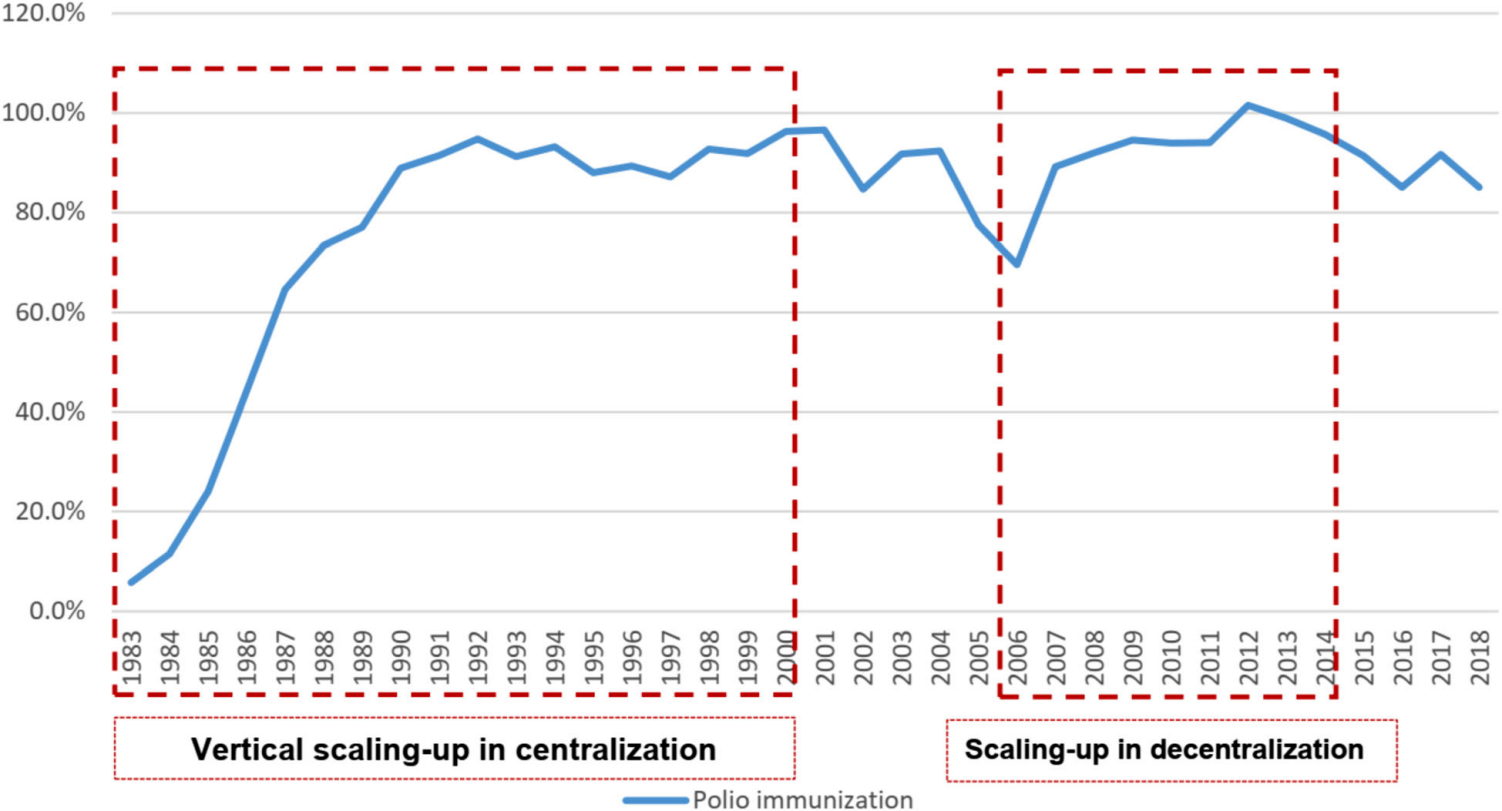
National polio immunization coverage 1983–2018. Source: Sub Directorate of Immunization, Ministry of Health (unpublished)

The multi-dimensional crisis (in 1998), an economic crisis followed with political crisis over the fallen of long-established government, and movement to the decentralized health system (1999–2000) influenced polio immunization coverage (Fig. 2). In the decentralization era, there was no longer direct command from the central level to the provincial level, as well as from provincial to the district level. Local governments became responsible for the delivery of immunization programs in their areas. Still, however, the central government remained responsible for additional immunization activities: providing vaccines, syringes and needles; technical assistance; developing guidelines; monitoring and evaluation; maintaining quality; and training. At the sub-national levels, EPI and VPD (Vaccine Preventable Diseases) surveillance were normally housed in the same section.

Although the coverage dropped around that time, additional activities, such as Supplementary Immunization Activities (SIA) s and National Immunization Day (NID), helped to boost polio immunization coverage after the outbreak in 2005. Thus, polio immunization coverage rebounded and increased incrementally in the decentralization era between 2006 and 2014. Afterward, in 2014, Indonesia received polio-free certification from SEARO. Independent organizations related to immunization emerged after the polio outbreak, such as Indonesian Technical Advisory Group on Immunization (ITAGI), National Certification Committee on Poliomyelitis Eradication (NCCPE), and Expert Review Committee (ERC).

In this paper, we identified two models of scaling up polio immunization coverage between 1983 and 2014. Program implementation in 2000–2004 was defined by its sustainability efforts and described elsewhere (Azizatunnisa' L, Cintyamena U, Mahendradhata Y, Ahmad RA. Ensuring sustainability of polio immunization in health system transition: lessons from the polio eradication initiative in Indonesia. Center for Tropical Medicine, Faculty of Medicine, Public Health and Nursing, Universitas Gadjah Mada; 2020. [unpublished report]).

As a global program, polio was rapidly scaled up in Indonesia and other countries. The polio immunization program in Indonesia started as vertical scaling up; the innovation was first institutionalized at the national level and replicated in the lower organization, before scaling up was continued in the decentralized era. Vertical scaling up has been described as an institutionalized program where the priority program is organized in upright management structures and service delivery arrangements [16]. Thus, vertical scaling up in Indonesia refers to the centralized health system where GPEI management was under direct control of the MoH until 1999.

**Fig. 3:**
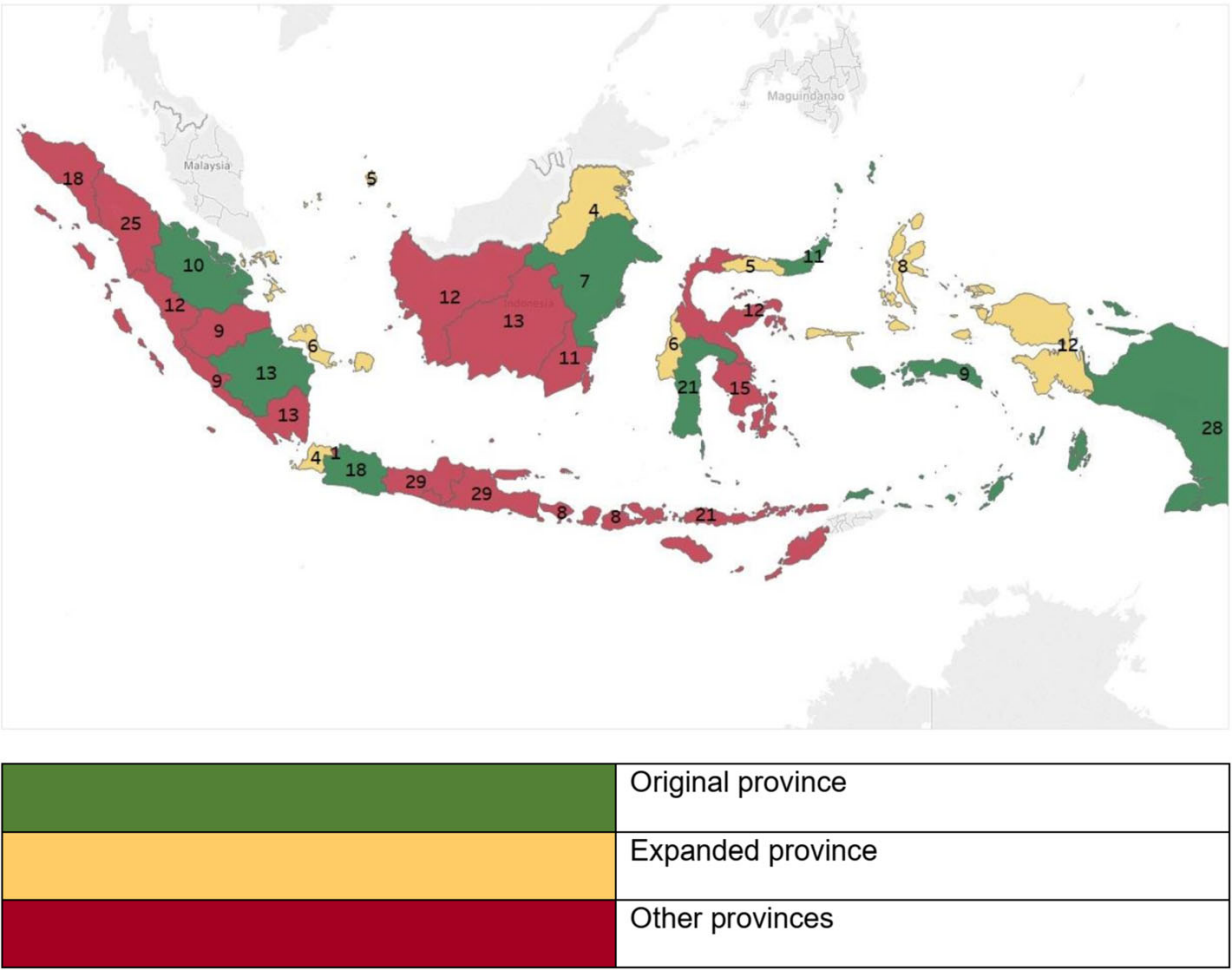
Expanded provinces and districts in decentralization era

**Fig. 4:**
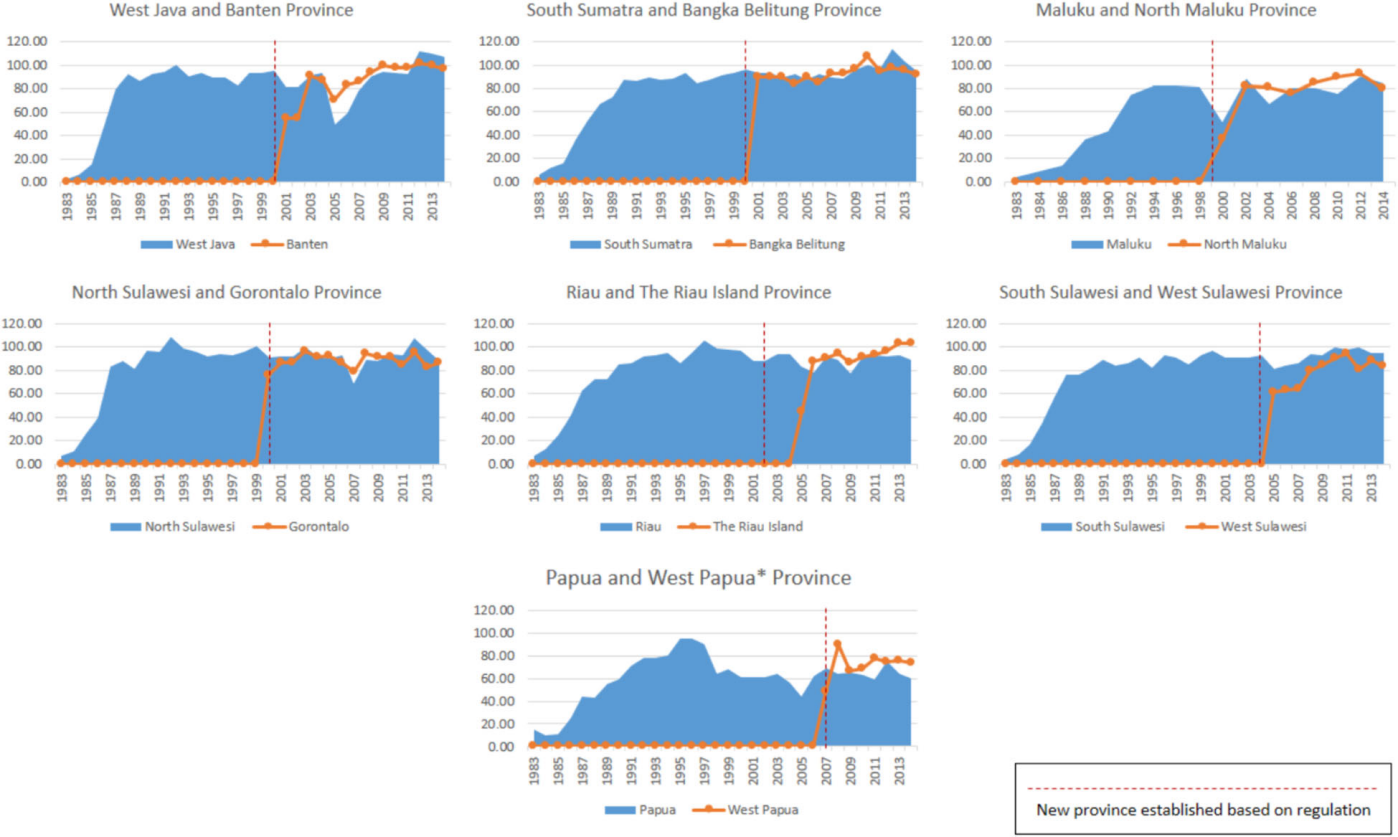
Percentage coverage of polio immunization in expanded provinces. Source: Sub Directorate of Immunization, Ministry of Health (unpublished)

The second scaling up model emerged in the decentralization era between 2006 and 2014. Ideally, the scaling up process that happened in decentralization should be classified as horizontal scaling up. A horizontal delivery approach is defined as a parallel process or replication of scaling up, where this model is delivered through regular infrastructure health facilities [16]. However, this was not the approach in Indonesia. In the decentralization era, no information showed that the scaling up happened due to the replication of the polio immunization program that occurred in expanded districts and provinces (Fig. 3). Hence, expanded activities occurred mainly due to the increasing number of sub-national government areas. Most expanded provinces show a lower polio coverage trend at the beginning of decentralization (Fig. 4). Although older provinces also experienced decreased coverage in the beginning decentralization era, but after 2005–2006 outbreak these provinces has faster coverage growth. Similar trend has been observed for other immunization programs.

Findings from both the centralized and decentralized era showed that polio immunization scaling up required collaboration of stakeholders and the community. Successful advocacy requests to national leaders made polio a priority program, with support from the non-health sector. Polio immunization was also well received by the community because of a massive movement towards community-based campaigns. Indonesia’s scaling up process can thus be summarized as the following: (i) planning; develop scaling up target output and innovation, (ii) identifying user organization and resource team, (iii) analyzing environment, (iv) deciding and implementing strategic planning, and (v) monitoring and evaluation.

### Challenges

From the survey, 54.8% of participants mention that external factors such as political, socio-economic, and technological factors were the most challenging facet in immunization activity. A total of 62.5% perceived that the social element was the most influential factor.

In the earlier stage of the polio immunization program, many *Puskesmas* did not conduct immunization service. This was due to issues with or unavailability of immunization infrastructure such as electricity, vaccine transport, or refrigeration equipment. Indonesia also has geographical disparity and topographical challenges which limited the availability of outreach activities. The availability of funding for this program was also limited until polio eradication became a program priority, together with mother-child health and the community nutrition program. Before Indonesia held the first NID in 1995, advocacy to the president had to be conducted several times. Lack of experience in conducting huge mass mobilization was also a challenge.

After no indigenous poliovirus was found, the barriers of this program were more related to the limited availability of outreach activities and cold chain maintenance, competing priorities in the decentralization era, limitation of funding, negative perception of immunization side-effects, and suspicion of vaccine ingredients which are considered forbidden by moslems (*haram*), despite awareness campaigns. From the health system perspective in the decentralization era, the barriers were decreasing commitment, changes in organizational structure, and the limitation of program monitoring. High staff turnover and the unsmooth process of transitioning from centralization towards decentralization are suspected to be the cause of a lack of continuity and accountability.

*“The turnover of health workers is also too fast. After they are trained, sometimes after 6 months or 1 year they have moved to other department.”* (Technical assistant, national level).

*“Maybe I will be said to blame the decentralization system, but if using decentralization system, we need more people and commitment from those people. If using centralization system, we only need one commitment from one person, and the commanding was easy.”* (Former manager, national level).

### Scaling up innovation

The main findings can be grouped into two categories: vertical scaling up in the centralization era and scaling up in the era of a decentralized health system. A summary of key findings from KII and the survey are presented in Table 1.

**Table 1:** Polio immunization scaling up elements

	Vertical Scaling Up in Centralization (1983–2000)	Scaling Up in Decentralization (2006–2014)
**Innovation**		
Strengthening service quality	Request support from NGO to strengthening logistics for immunization. Developed LAM, ASP, immunization supervision checklist	NIDs, SIAs, polio eradication was expanded to AFP surveillance and lab containment
New technology	To ensure cold chain: use oil-powered electricity, kerosene, and solar panel electricity	Switch from tOPV to bOPV, introduce Inactivated Poliovirus Vaccine. Immunization information system to trace childrens’ immunization status. Intensified research and development of non-porcine vaccines. Development vaccine combination.
Improving community-based interventions	Involving PKK. Integrated immunization in *Posyandu*; together with MCH and community nutrition	Involving community organization. Massive campaign by public figure
Services for underserved population	Additional frontline worker to improve coverage. “*Jurim*” or special officer for immunization	*Nusantara sehat* program, flying health care
New service delivery protocols, training, curricula, educational approaches	LAM continuous training at PHO, DHO, PHC, as well sub-district head. Delivery and training of ASP	National guidelines. Implementing minimum service standard at district level.
Financial, organizational, or managerial restructuring	Polio is integrated to immunization program and immunization was the only activity in polio eradication initiative. No special budget from government for polio immunization, so integrated to existing program.	Budget support from international and national NGO
Other capacity building interventions	Strong leadership from MoH stakeholder; both advocation and dissemination to upper level and to implementer in subnational level were great	
**Organization**	MoH and its networking, partner NGO, community	local government, MoH and its networking, FDA, Bio Farma, private providers, partner NGO, community, other ministries
**Resource team**	Policy maker in MoH, program managers, representatives of national and international NGO, researchers	Local stakeholder/ decision maker, policy maker from MoH, House of Representatives, Ministry of National Development Planning, Ministry of Home Affairs, program managers, representatives of national and international NGO, researchers, service providers
**Scaling up strategy**	Vertical scaling up	Diversification; through dissemination and advocacy to other sectors
**Environment**		
Policies and politics	Polio immunization program is new. Support from president and other sectors’ ministries.	However, after decentralization transition, political support from subnational government was not strong enough.
Bureaucracy	MoH through DGCG and DSHQ, also its networking conducted the program	Tiered managerial process at MoH and its networking. Local governments have their own authority to determine priority program. Regulation and legal protection are needed as basic element of health intervention activities.
Socio-economic and cultural conditions	Consists of numerous ethnicities and cultures. Indonesia was classified as low-income country.	Community pride in their own religions, cultures, and ethnicities. Socio-economic disparities.
People’s needs, perspectives, and rights	Community could obtain health service equally. People felt pride when participated.	Availability of choices in health service activities

#### Vertical scaling up in centralization era

At the initial stage, the main strategy was to prepare immunization infrastructure and to implement the program at the lowest level (*Puskesmas*). After EPI’s initiation program in 1977, the inclusion of the OPV became part of routine immunization in 1981 [17]. Polio was first introduced into routine immunization with 3 doses of OPV in 1983 and then in 1993 the number of doses increased to 4 [18].

Due to budget conditions, MoH requested financial support from external partners such as WHO, UNICEF, USAID, and Rotary. This support included hiring staff for polio-related immunization, surveillance and laboratory positions; developing and maintaining the infrastructure for vaccine production, procurement and logistics, surveillance and data reporting, and management; full financing of polio vaccines; purchasing physical assets (e.g., laboratory equipment, computers); and covering operational costs for mass immunization activities.

*“So, we need to build full chain infrastructure etc. The next challenge was service infrastructure”* (Program manager, national level).

With the Minister of Health decree in 1995, massive polio immunization campaigns emerged at the national level with two rounds of NIDs conducted in August and September, and repeated in 1996 and 1997. In addition to that, sub-NIDs were conducted by prioritizing several high-risk areas from 1998 to 2001 due to low polio immunization coverage, unsatisfactory AFP surveillance performance, and the high risk of wild poliovirus importation.

As mentioned by national level key informants, NID in the polio eradication program was the first public health activity in Indonesia that was supported with economic analysis, so this program was more acceptable to other sectors. These NIDs were supported by a massive campaign which involved other related ministries and institutions, such as Ministry of Home Affair, Minister of Development Planning, Food and Drug Administration, Bio Farma, and the Indonesia State Army at national level. At the community level, the involvement of *PKK* (women organization), Muhammadiyah, NU, Lions Club, Rotary Club, and other professional organizations assisted in increasing awareness, while some also supported immunization delivery.

*“In polio eradication (program) we (MoH) for the first time did cross-sectoral collaboration. Previously, we (MoH) worked alone.”* (Former stakeholder, national level).

#### Scaling up in decentralization era

NIDs continue to be conducted in the decentralization era. In 2002, the main purpose was to secure the goal of polio eradication in Indonesia after all the efforts. Supplementary immunization activities were conducted between 2005 and 2007 as a response to the polio outbreak. From 2009 to 2011, polio immunization campaigns were integrated with the measles crash program [19]. After that, polio immunization relied on the routine immunization program [20]..

To achieve milestone on scaling up polio program in decentralization era, the program focused more widely than just increasing polio immunization coverage through NIDs and SIA. Robust surveillance systems and lab containment were carried out to strengthen this program. Since 2002, additional staff were assigned as surveillance officers. AFP surveillance was extended to environmental surveillance in 2011. Moreover, there has also been new vaccine technology, such as the developments of non-porcine vaccine and vaccine combination. Additionally, legal protection in national immunization guidelines appeared to protect health workers from adverse events following immunization.

*“[After reforms], the condition change, we [government] were sued and the health workers were afraid to do immunization, asking for guarantee of legal protection.”* (Former stakeholder, national level).

### Implementation strategy

#### Local area monitoring (LAM) and area specific planning (ASP)

LAM is a monitoring tool to analyze immunization coverage based on target, time, and area [21]. This tool is developed by MoH and UNICEF and has been implemented since 1985. Two years after implementation, immunization coverage increased from 24.1 to 67.5%. Training of this new intervention was carried out by immunization managers at Provincial Health Office (PHO), District Health Office (DHO), PHC, and the sub-district head. Application of this tool led to competition among area administrators to achieve high immunization coverage. The implementation of an Area Specific Plan (ASP) for immunization further supported the efforts in achieving UCI.

*“Then the world heard that we had LAM, and then we were invited for the presentation in 92, on how we could reach UCI by using local area monitoring tools assisted by UNICEF”* (Program manager, national level).

#### Posyandu

*Posyandu,* (integrated health services post), is comprised of basic health activities from and for the community [22]. The services are carried out by lay cadres trained by a health worker from the *Puskesmas*. This activity first started by focusing on maternal and child health and family/community nutrition. In 1983, an immunization program was added.

*“Polio eradication will not succeed without strong immunization organization, and at the same period it was a period of public health development era through Posyandu … The aspect of community is very high”* (Program manager, national level).

## Discussion

The process of expanding the polio eradication effort in Indonesia was suitable to achieve the outcome of OPV immunization coverage status and AFP surveillance rates. Despite these indicators, other scaling up outcomes, such as cost-benefit and coverage on health system outcomes and provider outcomes, were not reported. Scaling up initiatives in other contexts usually reported patient/provider outcomes, occurred in LMICs, and were focused on infectious diseases [23]. Further research with more detailed questions can be used to discover these outcomes.

Findings presented in this manuscript are subject to a limitation of the used of OPV coverage only, while since 2016 polio immunization program in Indonesia rely on both OPV and IPV vaccination. Participant recruitment is also limited. There might be potential candidates who might not be interviewed due to geographical and time constrains. We also found difficulties in finding elderly participants who were involved at the beginning of program. Grey literatures data collection, especially government data and reports on the beginning of polio eradication initiatives, was also limited due to poor archiving of documents.

There were two models of scaling up in the Indonesia polio immunization program, i.e., vertical scaling up in the centralization era and scaling up in the decentralized health system. Vertical scaling up emerged before polio eradication became a national priority, and became more intense in 1995. It began with advocacy for adopting the innovation within the national program [9]. With NIDs as a special mass campaign, this effort became more massive. In the decentralized health system, the expansion did not specifically take place within expanded districts and provinces. Although coverage targets for the original provinces should be consistent with the accumulation of two new provinces, the mean coverage shows a decreasing trend. Although older provinces also experienced decreased coverage in the beginning decentralization era, but after 2005–2006 outbreak these provinces has faster coverage growth. Public awareness on polio immunization activity was decreased with no mass campaign program at the national level within 1998–2001. Besides, selected regional priorities had not been proven to be effective in maintaining the society. Government limitations on influencing the health program’s implementation at the sub-national level and upholding polio coverage during centralization and decentralization transition caused an increased risk of transmission. The 2005–2006 outbreak proved this. Fulfillment of health budget, which is less than 10% allocation, showed the importance and need for increasing local government ownership on health intervention during an era of decentralization [24]. Non vertical scaling up required strategic alternatives on disseminating innovation to different population groups, organized strategy, mobilization of resources, and monitoring and evaluation of the process, outcomes, and impact [9]. To gain high impact scaling up, there must be simple and cost-effective interventions and commitment of implementers and beneficiaries to work in tandem to build institutional capacity [24].

In Indonesia, early multi-sector commitment was gained through economic approaches. As mentioned by our key informant, the economic burden of vaccine-preventable diseases made multi-sector national stakeholders consider the importance of polio immunization for prevention. Although a previous study showed that centralization cannot be the only approach to stimulate community engagement, Indonesia’s previous health system demonstrated that centralization promoted Indonesia’s polio immunization program and multisector coordination with presidential support and approval [25].

In the decentralization era, the scaling up strategy by emphasizing polio immunization through *Puskesmas* was assumed to have the same effect for provincial and district stakeholders. The National Statistical Bureau data shows that 9 new provinces and 233 new districts were formed after decentralization, with massive expansion of an additional 144 local governments between 2000 and 2006 [26, 27]. Local stakeholders have their own authority in managing their regional budget and have a better understanding of the community condition; therefore, they should be better positioned to provide solutions and approaches to regional problems. Besides that, decentralization should accelerate service delivery in each area. Although the outcome effect varied, decentralization can improve health outcomes such as infant mortality rates, maternal mortality rates, and life expectancy [28]. Health outcomes could potentially be further enhanced through revitalization of *Posyandu* to be able to contribute more significantly, similar to the period before decentralization.

Although decentralization has been promoted to accelerate the service of delivery, case studies of the polio immunization program in Indonesia show success during the era of centralization. In the era of decentralization there were health system problems such as high staff turnover, budget limitations/ reduction, and lack of resources. Previous studies mention that the better health outcomes in the decentralization era will also be followed by higher expenditures [29, 30].

Furthermore, good program structure and program management will not be enough to ensure the program runs well. The previous success of the program occurred because of the commitment of MoH as the leading actors and the involvement of multisector organizations so it could reach the community. This commitment is based on good coordination and communication, as well as great leadership. Actors at sub-national (provincial and district) level conveyed good leadership from polio eradication stakeholders at the beginning of the implementation of this program, especially at early NIDs. Trust in employees, intense communication and flexibility to carry out programs make stakeholders and program managers at the sub-national level feel comfortable. Good communication within polio immunization program actors enables the activity to run well. Communication can increase the awareness of the involvement of stakeholders and communities. High awareness in the community will produce high participation as well.

At the beginning of the implementation of NIDs in Indonesia, many people assumed that it was a special campaign that needed a lot of resources. However, through NIDs, the community feels affiliated to the program and wants to regularly participate in polio activities. Unfortunately, at the same time, this also raises public perceptions that activities are difficult to put on or uphold without money. Strengthening previous findings, a global initiatives when combined with community movements can be an expression of local needs and politics, rather than the facilitation of information in support of predetermined goals [29]. Social engagement cannot only rely on planned activities from the central government, but must combine social mobilization from local and pragmatic activists in order to produce a successful movement [31].

## Conclusion

Vertical scaling up polio immunization program in Indonesia works fine during the earlier stage, yet requires linkages among system levels so that a program with a specific focus can be delivered. MoH’s decision to involve the president was critical since it promoted participation of non-health sector actors in the program. *Posyandu* played an important role, yet its revitalization after the reform-decentralization era has not been optimum. The polio immunization program must be owned by local stakeholders and decision makers.

## Data Availability

Dataset of polio immunization coverage is not publicly available due to the sensitivity of the data. Other generated and analyzed data are available from the corresponding author on reasonable request.

## Abbreviations

AFP: Acute Flaccid Paralysis
ASP: Area Specific Plan
DGDC: Directorate General of Disease Control and Prevention
DHO: District Health Office
DSHQ: Health Quarantine
EPI: Expanded Program on Immunization
ERC: Expert Review Committee
GPEI: Global Polio Eradication Initiative
KII: Key Informant Interview
ITAGI: Indonesian Technical Advisory Group on Immunization
LAM: Local Area Monitoring
LMIC: Low-Middle Income Country
NCCPE: National Certification Committee on Poliomyelitis Eradication
NID: National Immunization Day
NPLN: National Polio Lab Network
MoH: Ministry of Health
OPV: Oral Poliovirus Vaccine
PHC: Primary Health Care
PHO: Provincial Health Office
SEARO: South East Asia Region Office
SIA: Supplementary Immunization Activities
UCI: Universal Child Immunization
VPD: Vaccine Preventable Diseases
WHO: World Health Organization
